# Genetic variation in the adiponectin receptor 2 (*ADIPOR2*) gene is associated with coronary artery disease and increased ADIPOR2 expression in peripheral monocytes

**DOI:** 10.1186/1475-2840-9-10

**Published:** 2010-02-23

**Authors:** Iosif Halvatsiotis, Panayoula C Tsiotra, Ignatios Ikonomidis, Anastasios Kollias, Panagiota Mitrou, Eirini Maratou, Eleni Boutati, John Lekakis, George Dimitriadis, Theofanis Economopoulos, Dimitrios T Kremastinos, Sotirios A Raptis

**Affiliations:** 1 Hellenic National Centre for the Research, Prevention and Treatment of Diabetes Mellitus and its Complications (HNDC), Athens, Greece; 2 2nd Department of Internal Medicine, Research Institute and Diabetes Centre, Medical School, University of Athens, "Attikon" University General Hospital, Athens, Greece; 3 2nd Department of Cardiology, Medical School, University of Athens, "Attikon" University General Hospital, Athens, Greece

## Abstract

**Background:**

Adiponectin is an adipose tissue secreted protein known for its insulin sensitising and anti-atherogenic actions. To this date two adiponectin receptors have been discovered, adiponectin receptor 1 (ADIPOR1) and adiponectin receptor 2 (ADIPOR2). The aim of this study was to investigate the association of *ADIPOR2 *gene variations with coronary artery disease (CAD).

**Methods:**

Eight common single nucleotide polymorphisms (SNPs) spanning the entire *ADIPOR2 *locus were chosen to perform association studies with anthropometric and metabolic parameters in a Greek population. They were classified as either CAD (stenosis >50% in at least one main vessel) or non-CAD individuals in accordance with coronary angiography data.

Genotyping was performed using a microsphere-based suspension array and the Allele Specific Primer Extension (ASPE) method. Expression of ADIPOR2 protein and mRNA in circulating CD14^+ ^monocytes were determined using flow cytometry and real time Polymerase Chain Reaction assays respectively.

**Results:**

There was a significant difference in the distribution of genotypes of polymorphism rs767870 of *ADIPOR2 *between CAD and non-CAD individuals (p = 0.017). Furthermore, heterozygotes of the rs767870 polymorphism had significantly lower Flow Mediated Dilatation (FMD) values, higher values of Intima-Media Thickness (IMT) and increased ADIPOR2 protein levels in peripheral monocytes, compared to homozygotes of the minor allele after adjustment for age, sex, waist to hip ratio and HOMA.

**Conclusions:**

Our findings suggest that variants of *ADIPOR2 *could be a determinant for atherosclerosis independent of insulin resistance status, possibly by affecting ADIPOR2 protein levels.

## Background

Adiponectin is a protein secreted from adipocytes released in the circulation of human healthy subjects at relatively high levels [1-4]. Plasma adiponectin levels have been reported as decreased in states of obesity, type 2 diabetes and coronary artery disease [5-8]. Adiponectin exerts its insulin-sensitising effects in the liver by suppressing gluconeogenesis and in the skeletal muscle by enhancing fatty acid oxidation [9]. Furthermore, adiponectin exhibits anti-inflammatory and atheroprotective actions in various tissues by suppressing the expression of vascular adhesion molecules and scavenger receptors, reducing the expression of the inflammatory cytokine TNF-α, raising NO production and suppressing the proliferation and migration of smooth muscle cells [10-14].

To this date, two receptors have been identified that mediate adiponectin's actions in fatty-acid oxidation and glucose uptake, namely ADIPOR1 and ADIPOR2 [15]. Both receptors are almost ubiquitously expressed in most tissues, albeit at different levels, and studies aimed at their mRNA and protein expression levels in various insulin resistant states have produced inconclusive results [16-18]. It has been reported that the expression of these receptors is either induced or reduced in adipose and muscle tissues from obese and insulin resistant subjects [19,20]. Furthermore, it was recently shown that monocytes from overweight and obese individuals with type 2 diabetes compared to normal-weight controls have an impaired expression of adiponectin receptors [21].

ADIPOR2 is a cell-surface receptor abundantly expressed in skeletal muscle and liver, serving as a receptor for both globular and full-length adiponectin. Its protein expression has been demonstrated to be either up-regulated in adipose tissue from insulin resistant women with polycystic ovarian syndrome, or down-regulated in monocytes from overweight/obese patients with type 2 diabetes [19,21]. Similarly, its mRNA expression in skeletal muscle and adipose tissues from obese, insulin resistant or type 2 diabetic patients follows the same inconclusive results [17,18]. The *ADIPOR2 *gene is located on chromosome 12p13.33, consisting of eight exons. Single nucleotide polymorphisms (SNPs) of the *ADIPOR2 *have been associated with either insulin resistance or hepatic fat accumulation in various populations [22-29], albeit not in all studies [30-33]. Nevertheless, the role of genetic variants of *ADIPOR2 *in coronary artery disease has not been studied yet.

In this study, we investigated the association between eight common single nucleotide polymorphisms of the *ADIPOR2 *gene with the presence of coronary artery disease and its protein expression from human peripheral monocytes from the same individuals.

## Methods

### Subjects

Our study analysis consisted of 68 patients from the Greek population with cardiovascular risk factors, who were screened for the existence of chronic stable CAD. All individuals underwent elective coronary angiography. Case subjects (n = 40) were patients who had angiographic evidence of stenosis > 50% in at least one major coronary artery (CAD). Control subjects (n = 28) were people without coronary stenosis at angiography (non-CAD). Subjects with acute myocardial infarction, systemic inflammatory diseases, malignancies, renal failure (creatinin > 1.5 mg/dl), heart failure and severe obesity with body mass index (BMI) > 35 were excluded from our study.

All patients gave their written informed consent and the study protocol was approved by the Scientific and Ethics Committee of Attikon University General Hospital. All patients were of a stable weight and had been on a normal isocaloric diet with normal physical activity during the previous four months. None of the patients were taking thiazolidinedione medication. Waist and hip circumferences were measured and the waist to hip ratio (WHR) was calculated. BMI was calculated as the ratio of weight (Kg) to height (m^2^). All patients were subjected to Intima-Media Thickness (IMT) assessment in common carotids and in carotid bulbs as an index of atherosclerosis, using B-mode ultrasound imaging (Vivid 7 General Electric Horten, Norway), as previously described [34] and Flow Mediated Dilatation (FMD) assessment of the brachial artery as an index of endothelial dysfunction [35].

The coronary angiography was followed by a 2-hour oral glucose tolerance test (OGTT) two weeks later. Insulin resistance status in the fasting state was calculated by the homeostasis model assessment index (HOMA) [36], whilst post-glucose insulin sensitivity was calculated by the Matsuda index [37]. Fasting blood was drawn at the beginning of the OGTT and plasma was kept at -80°C for measurement of adiponectin levels. Fasting triglycerides, as well as total and high-density lipoprotein (HDL) cholesterol were determined by an ILAB Analyser (Instrumentation Laboratory SpA, Italy). Plasma glucose levels at fasting and during the OGTT were analysed on a Falcor 300 chemical analyser (Menarini diagnostics, Italy) and their corresponding insulin levels were measured by IRMA (INSI-CTK, Dia Sorin, Italy).

### Genomic DNA isolation

**Figure 1:**
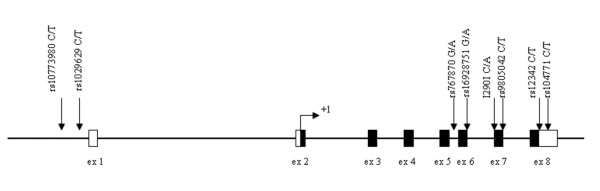
**Schematic representation of the *ADIPOR2 *gene**. The polymorphisms studied are represented by vertical arrows. Empty boxes denote exons and black boxes denote sequences translated into protein. +1 represents the ATG translation start site.

Fasted blood was collected from all individuals and genomic DNA was extracted from the buffy coat according to the manufacturer's instructions (Flexigene DNA Kit, Qiagen, Chatsworth, CA, USA). The quality and the integrity of the DNA samples was determined by standard ethidium-bromide agarose gels and quantification was performed spectrophotometrically (UV/VIS Spectrometer Lambda Bio10, Perkin Elmer, USA). The following common single nucleotide polymorphisms were examined lying either in the promoter [rs10773980 C/T and rs1029629 C/T], the intron 5 [rs767870 A/G], the exon 6 [rs16928751 G/A], the exon 7 [rs9805042 C/T and I290I C/A] and the exon 8 [rs12342 C/T and rs1044771 C/T] of *ADIPOR2 *(Fig. 1).

### Genotyping of ADIPOR2 polymorphisms using the Allele Specific Primer Extension (ASPE)

The Allele Specific Primer Extension assay and xMAP technology were used to genotype our samples. This technique is a solution-based sequence-specific enzymatic reaction that determines the target SNPs. It involves the incorporation of a specific capture sequence, named Allele Specific Primer Extension (ASPE) that carries the major or the minor nucleotide of the SNP at its 3' -end [38,39]. Therefore, two ASPE primers are needed for every SNP in order to resolve each possible allele pair. ASPE primers also carry a tag-sequence which hybridises to a complementary sequence called anti-tag, in the surface of the polystyrene microspheres (xMAP microspheres), which are internally dyed with fluorophores.

**Figure 2:**
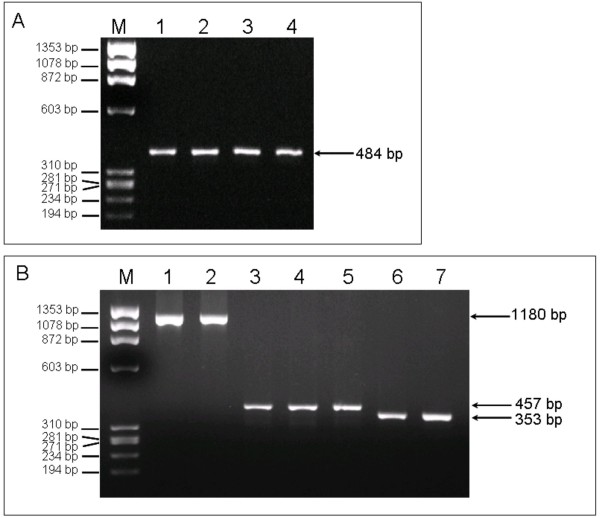
**Representative PCR reactions showing the PCR specific bands for all polymorphisms studied of the *ADIPOR2 ***(A). Detection of the PCR specific bands for polymorphism rs767870, lanes 2 and 3 show the PCR specific bands amplified from human genomic DNA from two CAD individuals, while lanes 4 and 5 show the PCR bands amplified from human genomic DNA from two non-CAD individuals. (B) Detection of the PCR specific bands for polymorphisms: rs10773980 (lane 1), rs1029629 (lane 2), rs16928751 (lane 3), I290I (lane 4), rs9805042 (lane 5), rs12342 (lane 6) and rs1044771 (lane 7). Arrows show the base pair size of the various PCR products. M: ΦX174-HaeIII digest

Various PCR products encompassing the SNPs of interest, ranging in size from 353 to 1180 bp, were generated through standard PCR reactions from human genomic DNA, using Platinum Taq Polymerase (Invitrogen, Carlsbad, CA, USA) in a GeneAmp 9600 thermocycler (Perkin Elmer Inc, Massachusetts, USA). An aliquot from the PCR reactions was run on 1.5% agarose gels and visualised with ethidium bromide staining. Product lengths were estimated using a standard molecular weight marker (ΦX174-HaeIII digest, NEB, UK) (Fig 2A and 2B) and subsequently the PCR products were treated with exo-SAP mix (2:1) (Exonuclease I:Shrimp Alkaline Phosphatase, USB Europe GmbH, Germany) at 37°C for 30 minutes in order to remove unconsumed dNTPs and primers remaining in the mixture, followed by inactivation at 80°C for 15 minutes. Consequently the treated PCR products were combined with ASPE primers and extension took place with the use of biotin-14-dCTP and Platinum Tsp DNA Polymerase (Invitrogen, Carlsbad, CA, USA). Extension occurs only if the 3' nucleotide of the ASPE primer is complementary to the template DNA. The conditions for the ASPE reaction were as follows: denaturation at 96°C for 2 minutes, amplification for 40 cycles at 95°C for 30 seconds, 54°C for 30 seconds and 74°C for 60 seconds, followed by one cycle at 4°C. The subsequent hybridisation of the anti-tag microsphere to the biotinilated ASPE-tag PCR product took place at 37°C for 60 minutes, after a short denaturation cycle at 95°C for 90 seconds.

**Figure 3:**
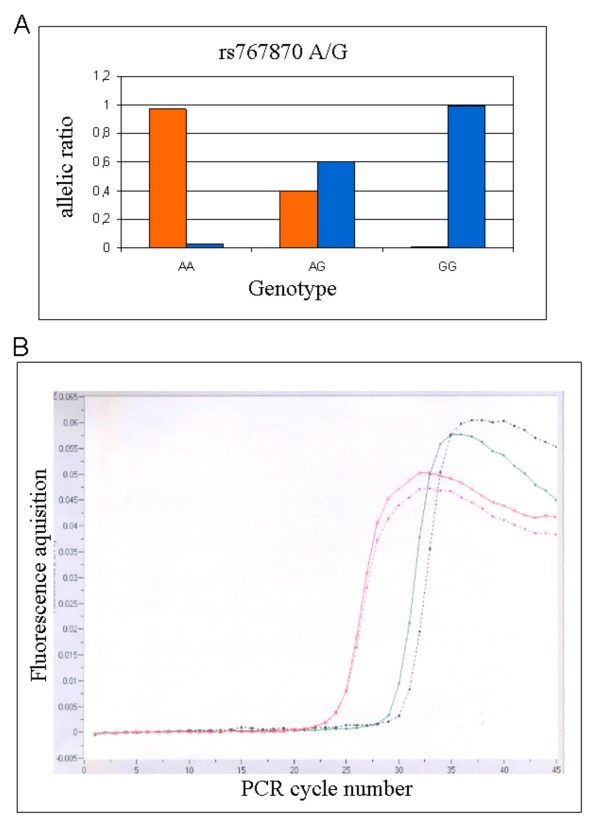
**Diagrammatic representation of: (A) the genotyping results for rs767870 polymorphism of *ADIPOR2 *using the Allele Specific Primer Extension assay and the xMAP technology and (B) the detection of the relative *ADIPOR2 *mRNA levels using the real-time PCR assay**. A. Bars represent the two alleles of the rs767870 polymorphism (G allele: blue bars, A allele: orange bars). The results show three different individuals: on the left, an individual homozygote for the A allele (AA genotype), in the centre, a heterozygote carrying both alleles (AG genotype) and on the right side, a homozygote for the G allele (GG genotype). B. A quantification analysis screen from a typical real-time PCR run, showing the fluorescence acquisition for the ADIPOR2 (green and black curves) and the β-actin (red and pink curves) mRNA levels on the y axis and the number of cycles on the x axis. Results are shown for two different individuals, a CAD (green and red curves) and a non-CAD individual (black and pink curves).

Finally, a fluorescent-labelled reporter molecule (Streptavidin-R-phycoerythrin, 1 mg/ml, Molecular Probes, Invitrogen, Carlsbad, CA, USA) binds to the biotinilated hybrid ASPE microsphere product. This coupling of the reporter molecule quantifies the biomolecular interaction that occurs at the microsphere surface. The reaction can finally be analysed on a Luminex 200 instrument (Luminex Corp, TX, USA). Precision fluidics aligns the microspheres in single file and passes them through the lasers, one at a time. One laser excites the reporter molecule bound to the microsphere surface and the second laser excites the microsphere fluorophores. Reactions are measured with fluorescent intensity and reported in real time. The fluorescent signals generated for each microsphere bead population were used to assess the genotype in each sample. The median fluorescent intensities (MFI) were used to calculate allelic rations for each allele as follows:

To be homozygous for a particular allele, the allelic ratio must be at least 0.75. For heterozygous samples each allele must have a ratio between 0.25 and 0.75. An allele with a ratio of 0.25 or less is considered negative i.e. not present. Representative samples results are shown in figure 3A.

### Determination of ADIPOR2 mRNA and protein expression

Peripheral monocytes were isolated from mononuclear cells using a Magnetic Cell-Sorting technique and the CD14 Human MicroBeads (Miltenyi Biotec, Gladbach, Germany). The purity of the magnetically isolated CD14^+ ^monocytes was determined after staining the cells with a CD14 PE-CD45 FITC antibody (BD Biosciences, San Jose, CA, USA).

Total RNA was extracted from CD14^+ ^monocytes using the Tripure Isolation Reagent (Roche Diagnostics GmbH, Mannheim, Germany). Reverse transcription of 1 μg of total RNA was performed in all samples with the Transcriptor First Strand cDNA Synthesis Kit using random hexamer primers, according to the manufacturer's instructions (Roche Diagnostics GmbH, Mannheim, Germany).

A quantitative real-time PCR using fluorescent-labelled hybridisation probes was developed for detecting relative *ADIPOR2 *mRNA levels, in human CD14^+ ^monocytes, using the calibrator-normalised standard curve and the LightCycler Relative Quantification 1.0.1 Software, in a spectrofluorometric thermal cycler (LightCycler, ROCHE, Manheim, Germany), as previously described [40]. The human housekeeping gene of β-actin was used as standard for normalisation. Hybridisation specific primers and fluorescent-labelled hybridisation specific probes for the genes were designed and manufactured by the TIM-MOLBIOL (Berlin, Germany). Hybridisation primers and probes for the target and reference genes were as follows:

Primers were chosen to lie between exons whenever possible and the analysis was performed with the programme OLIGO 6.0 (Molecular Biology Insights, Cascade, USA).

For quantifying the relative expression levels of *ADIPOR2 *mRNA in the various samples, we ran the target (AdipoR2) and the reference (β-actin) gene of each sample along with the calibrator cDNA, using the Second Derivative Maximum Method with the Arithmetic baseline adjustment for the determination of the various crossing points (Fig 3B). Samples were run in duplicates or triplicates and results are expressed in arbitrary units.

Surface adiponectin receptor ADIPOR2 was determined after staining the isolated cells ('buffy'coat) with a suitable antibody (Alpha Diagnostic International, San Antonio, USA). Since the antibody was not fluorochrome conjugated, it was labelled with the Zenon™ Alexa Fluor^® ^488 Rabbit IgG labelling kit (Invitrogen, Carlsbad, CA, USA). Cells were incubated for 30 minutes with Alexa Fluor^® ^488-conjugated immunoglobulin in a ratio 1 × 10^6^cells μg^-1 ^of immunoglobulin under mild constant shaking. The monocyte fraction was simultaneously stained with anti-CD14-PE monoclonal antibody (BD Biosciences, San Jose, CA, USA). The specificity of the antibody used was evaluated by staining cells with isotype control suitable for each antisera and the blockage of Fc-receptors prior to staining.

Two-colour flow cytometric analysis was performed on a BD FACSCalibur 4 colour flow cytometer (BD Biosciences) equipped with two air-cooled lasers: a 15 mW Argon laser (488 nm) and a Red Diode laser (632 nm). Data acquisition and analysis were performed using the BD CELL Quest Pro software (BD Biosciences). Results are expressed in mean fluorescence intensity arbitrary units (MFI AU).

### Adiponectin plasma levels

Circulating levels of adiponectin were measured in the plasma of all subjects, using a high sensitivity multiplex assay (xMAP technology) and fluorescent-labelled microsphere beads (HCVD1-67AK Lincoplex kit, Millipore Corp., MA, USA), in a LUMINEX 200 instrument (Luminex Corp, TX, USA). Sensitivity of the assay was 56.0 pg/ml, intra-assay and inter-assay coefficients of variation were 9.2% and 15.9%, respectively. All samples were diluted 1:100 and measured in duplicate. Samples that could not be detected were measured again using a higher dilution.

### Statistical analysis

Statistical analysis was performed using the SPSS version 14.0 software (Chicago IL, USA). Deviation of SNPs from the Hardy-Weinberg equilibrium was performed using the chi-square test and the Hardy-Weinberg equilibrium calculator [41]. If genotype frequencies for a particular SNP differ from those expected under equilibrium, the Hardy-Weinberg principle is being violated and is therefore not safe to try to associate this SNP with coronary artery disease. Distribution of alleles was compared using the χ^2 ^and the level of significance adopted was p < 0.05. The whole population odds ratio associated with genotypes was calculated by binary logistic regression analysis. Normal distribution was tested with the Kolmogorov-Smirnov test and logistic transformations of variances to achieve normal distribution were used when needed. The differences in various variables between genotypes were evaluated with the univariate ANOVA, adjusted for age, sex, BMI and HOMA. Kruskal-Wallis test was also used when homogeneity of variances were not met. Non-parametric Mann-Whitney and 2-independent samples t-tests were used accordingly for analysis. Data are expressed as means ± SEM.

## Results

### General characteristics of the subjects

The clinical and metabolic characteristics of the population studied (CAD and non-CAD) are shown in table 1. There were no significant differences between the two groups regarding age, weight, BMI, fasting and two-hour glucose and insulin levels, insulin resistance indices (HOMA and Matsuda), lipid levels (total cholesterol, LDL, HDL), systolic and diastolic blood pressure, endothelial dysfunction (FMD) and the circulating adiponectin (Table 1). However, CAD patients had higher WHR and IMT values compared to control subjects (Table 1). Furthermore, CAD subjects had higher *ADIPOR2 *mRNA levels, while they did not differ in *ADIPOR2 *protein expression, from peripheral CD14^+^monocytes, compared to controls.

**Table 1:** Anthropometric and metabolic characteristics of the people studied

	NonCAD (n = 28)	CAD (n = 40)	p
***Anthropometric characteristics***			
Age (years)	56.61 ± 1.47	59.80 ± 1.34	NS
Body weight (kg)	80.28 ± 2.09	83.69 ± 1.51	NS
BMI (Kg/m^2^)	28.12 ± 0.51	28.53 ± 0.49	NS
WHR	0.93 ± 0.01	0.99 ± 0.01	**0.001**
***Metabolic characteristics & Indices***			
Fasting plasma glucose (mg/dl)	106.76 ± 6.19	113.29 ± 4.5	NS
Two-hour plasma glucose (mg/dl)	162.81 ± 15.30	199.35 ± 12.79	NS
Fasting plasma insulin (μU/ml)	14.39 ± 1.84	13.79 ± 1.15	NS
Two-hour plasma insulin (μU/ml)	100.09 ± 12.74	135.41 ± 22.53	NS
HOMA index	3.80 ± 0.58	4.68 ± 0.69	NS
Matsuda index	2.84 ± 0.24	2.68 ± 0.25	NS
Total cholesterol (mg/dl)	193.26 ± 8.55	183.32 ± 5.85	NS
LDL (mg/dl)	128.00 ± 8.68	122.71 ± 4.88	NS
HDL (mg/dl)	49.29 ± 2.62	45.97 ± 1.97	NS
Trigycerides (mg/dl)	114.46 ± 10.04	133.39 ± 10.95	NS
Systolic blood pressure (mm Hg)	122.32 ± 2.94	128.06 ± 2.57	NS
Diastolic blood pressure (mm Hg)	76.25 ± 1.76	78.89 ± 1.34	NS
IMT(mm)	0.83 ± 0.05	1.09 ± 0.05	**0.001**
IMT bulb (mm)	1.13 ± 0.15	1.45 ± 0.08	NS
FMD (%)	8.59 ± 1.28	7.01 ± 0.81	NS
Adiponectin (μg/ml)	13.01 ± 1.09	13.18 ± 1.30	NS
ADIPOR2 mRNA (AU)	0.81 ± 0.06	1.2 ± 0.16	**0.035**
ADIPOR2 protein (MFI)	82.97 ± 9.83	65.60 ± 4.68	NS

Data are given as means ± SEM. BMI: Body Mass Index, LDL: Low Density Lipoprotein, HDL: High Density Lipoprotein, IMT: Intima Media Thickness; FMD: Flow Mediated Dilatation

### Allele frequencies of ADIPOR2 polymorphisms

The allele frequencies of the investigated polymorphisms of the *ADIPOR2 *are shown in table 2. Only rs767870 and rs1044771 polymorphisms were found to be in Hardy-Weinberg equilibrium, (p < 0.05, χ^2 ^< 3.83). Thus we excluded from further analysis, any polymorphism that deviated from Hardy-Weinberg equilibrium.

**Table 2:** Allele frequencies of *ADIPOR2 *gene polymorphisms in the Greek population studied

*Polymorphism*	*Major/minor allele*	*allele frequencies*
rs10773980 C/T	C/T	C: 0.507, T: 0.492

rs1029629 C/T	C/T	C: 0.662, T: 0.338

rs767870 A/G	A/G	A: 0.801 G: 0.199,

rs16928751 G/A	G/A	G: 0.853, A: 0.147

I290I C/A	C/A	C: 0.889, A: 0.111

rs9805042 C/T	C/T	C: 0.632, T: 0.368

rs12342 C/T	C/T	C: 0.507, T: 0.493

rs1044771 C/T	C/T	C: 0.463, T: 0.537

### Association of rs767870 SNP with coronary artery disease

We subsequently investigated the distribution of genotypes for the rs767870 and rs1044771 polymorphisms between the two populations and their possible association with coronary artery disease (Table 3). There was a significant difference in the distribution of alleles of rs767870 polymorphism of *ADIPOR2 *between CAD and non-CAD individuals (p = 0.017).

**Table 3:** Associations between polymorphisms of *ADIPOR2 *and the risk of coronary artery disease

SNPs	Major/minor allele	genotype	CAD (%)	Non CAD (%)	p^a^	OR (95% CI) p^b^
rs767870	A/G	AA	70	64.2	0.017	0.479 (0.465-5.120)
		AG	30.0	17.9		0.999
		GG	0.0	17.9		
rs1044771	C/T	CC	22.5	10.7	0.510	0.557 (0.317-8.454)
		CT	50.0	67.9		0.355
		TT	27.5	21.4		

Genotype distributions are shown as per cent (%). ^a ^p-value according to chi-square test, ^b ^p-value according to binary logistic regression analysis, OR: Odds ratio, 95% confidence interval (CI)

No significant difference was found in the distribution of alleles for the rs1044771 polymorphism of *ADIPOR2 *between the two groups (Table 3).

**Figure 4:**
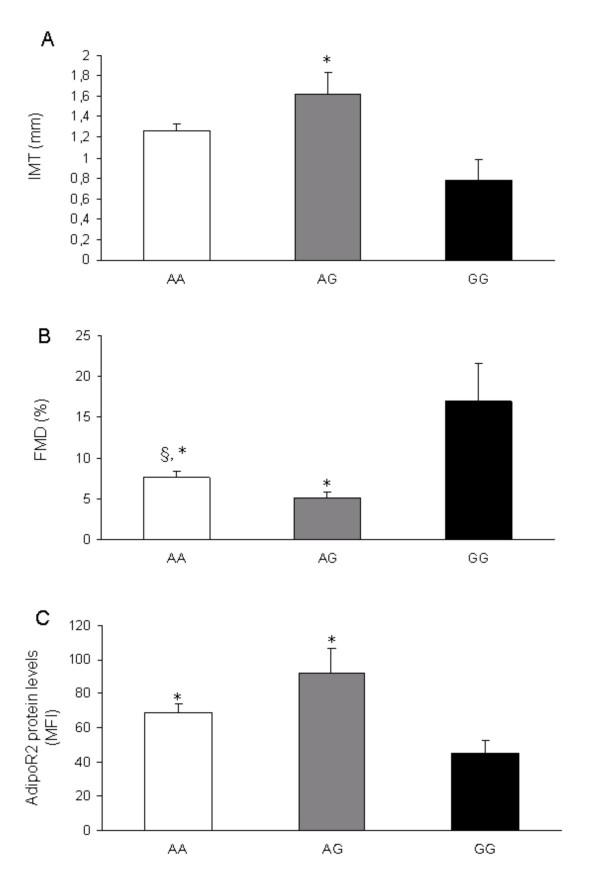
**Genotype effects of the SNP 767870 of *ADIPOR2 *on the: (A) IMTbulb measurement, (B) FMD measurement and (C) ADIPOR2 protein levels from CD14+ monocytes**. Data are means ± SEM. Differences between genotype groups were analysed by ANOVA adjusted for age, sex, BMI, WHR and HOMA (A and B, p = 0.04 and p = 0.002 respectively) and ANOVA adjusted for age, sex, WHR and HOMA (C, p = 0.05) * p < 0.05 vs GG homozygotes §p < 0.05 vs AG heterozygotes.

Using binary logistic regression analysis, the risk for CAD for AG heterozygotes of the rs767870 polymorphism was 50% higher compared to AA homozygotes of the same polymorphism, although not to a significant degree (OR = 1.543 and 95% CI: 0.465-5.120) (Table 3). Furthermore, the marker rs767870 of *ADIPOR2 *was most significantly associated with waist circumference, IMT and FMD measurements in all individuals (Table 4). To be more specific, AA homozygotes and AG heterozygotes of the rs767870 had significantly higher IMTbulb and lower FMD values compared to GG homozygotes (1.62 ± 0.21 mm and 5.09 ± 0.76% vs 1.26 ± 0.07 mm and 7.62 ± 0.74%, p = 0.025 and p = 0.002, adjusted for age, sex, BMI, WHR and HOMA, respectively) (Table 4 and Fig.4A and 4B).

**Table 4:** Anthropometric and metabolic characteristics of the study population divided according to the genotypes of rs767870 of the *ADIPOR2*

rs767870	A/A	A/G	G/G	p	p
SEX (male/female)	40/6	15/2	3/2	0.25^a^	
Age (years)	58.50 ± 1.29	59.53 ± 1.88	54.80 ± 2.48	0.54	
Weight (Kg)	81.73 ± 1.43	86.25 ± 2.74	75.10 ± 3.58	0.078	
Height (cm)	1.70 ± 0.01	1.71 ± 0.01	1.63 ± 0.01	0.056	
BMI (Kg/m^2^)	27.97 ± 0.40	29.46 ± 0.82	28.18 ± 1.09	0.198	
Waist circumference (cm)	98.23 ± 1.39	103.37 ± 2.55	91.6 ± 3.88	**0.040**	0.067^b^
Hip circumference (cm)	101.52 ± 1.01	104.75 ± 2.34	100.20 ± 2.99	0.278	
WHR	0.96 ± 0.01	0.99 ± 0.01	0.91 ± 0.03	0.128	
IMT (mm)	1.01 ± 0.05	1.02 ± 0.09	0.65 ± 0.09	**0.028^c^**	0.053^d^
IMT bulb (mm)	1.26 ± 0.07	1.62 ± 0.21	0.78 ± 0.21	**0.019**	**0.025^e^**
FMD (%)	7.62 ± 0.74	5.09 ± 0.76	16.97 ± 4.54	**0.0001**	**0.002^e^**
HOMA	4.02 ± 0.63	4.42 ± 0.47	5.09 ± 2.81	0.887	
Matsuda	2.87 ± 0.24	2.31 ± 0.23	3.12 ± 0.72	0.345	
Adiponectin (μg/ml)	12.42 ± 0.73	15.82 ± 2.86	10.19 ± 1.24	0.168	
AdipoR2 mRNA (AU)	1.02 ± 0.12	1.16 ± 0.23	0.83 ± 0.10	0.732	
AdipoR2 protein (MFI)	69.17 ± 4.80	92.03 ± 14.41	45.20 ± 7.70	**0.039**	**0.008^f^**

Data are means ± SEM, ^a^chi square test, ^b^ANOVA for comparison among all three genotype groups adjusted for age and sex, ^c^ANOVA for comparison among all three genotype groups after logarithmic transformation of values, ^d^ANOVA for comparison among all three genotype groups adjusted for age and BMI, after logarithmic transformation, ^e^ANOVA for comparison among all three genotype groups adjusted for age, sex, BMI, WHR and HOMA, ^f^ANOVA for comparison among all three genotype groups adjusted for age, sex, WHR and HOMA.

We then investigated the possibility that the association of rs767870 polymorphism with markers of endothelial dysfunction and atherosclerosis may be mediated through an effect on gene expression. In peripheral CD14^+ ^monocytes, carriers of the major A allele (homozygotes and heterozygotes) of rs767870 polymorphism had higher levels of ADIPOR2 protein expression compared to homozygotes of the minor allele (69.17 ± 4.80 MFI and 92.03 ± 14.41 MFI vs 45.20 ± 7.70 MFI, p = 0.008, after adjustment for age, sex, WHR and HOMA) (Table 4 and Fig. 4C).

## Discussion

We have demonstrated for the first time that a sequence variant in the intron 5 of the *ADIPOR2*, rs767870 among the eight studied, is associated with cardiovascular disease in our population of Greek individuals. Furthermore, genotypes from this variant are associated with higher IMT and lower FMD values in the same population, while they exhibit higher ADIPOR2 protein levels in circulating monocytes.

*ADIPOR1 *and *ADIPOR2 *are considered promising genes for type 2 diabetes, the metabolic syndrome and its complications, such as cardiovascular disease [42]. The *ADIPOR2 *is located on chromosome 12p13.31 and comprises of eight exons. Various studies have associated genetic variants of the *ADIPOR2 *locus with insulin resistance and the traits of the metabolic syndrome [21-29], whilst several others have failed to confirm this [30-33]. In particular, *ADIPOR2 *variants have been strongly associated with type 2 diabetes in the Old Amish Order and in a Chinese population [22,24]. Other genetic variations of *ADIPOR2 *have been associated with decreased triglyceride levels in Mexicans with insulin resistance and in Germans with metabolic syndrome [27,28].

We chose to study *ADIPOR2 *because the effects of *ADIPOR2 *gene variants in cardiovascular disease have not yet been examined. It has been demonstrated that at least for *ADIPOR1*, sequence variants in its 3'-region, are significant determinants of cardiovascular risk in type 2 diabetes [43]. Moreover in the same study, the authors have found that haplotypes of the above variants are also associated with lower *ADIPOR1 *mRNA levels in mononuclear cells and adipose tissue biopsies.

In our study, we have demonstrated that carriers of the major allele of the rs767870 polymorphism of the *ADIPOR2 *are associated with 78% higher ADIPOR2 protein expression in circulating monocytes, compared to homozygotes of the minor allele, independent of age, gender, obesity measures and insulin resistance. Furthermore, these same individuals compared to minor allele homozygotes, have 56% and 83% higher IMT values at both common carotids and carotid bulbs respectively, while at the same time they show 67% lower FMD values, independent of age, gender, obesity measures and insulin resistance. It has been shown that the reduction of FMD increases the risk of coronary artery disease [44], whilst increments in the IMT measurements are associated with atherosclerosis progression [45]. In line with this, our findings could suggest that individuals carrying the A allele of the rs767870 polymorphism of *ADIPOR2 *are prone to atherosclerotic events that could possibly be mediated via increased *ADIPOR2 *expression.

The mechanisms through which variations in the *ADIPOR2 *could influence atherosclerosis and cardiovascular disease are only hypothetical at the moment. The rs767870 polymorphism lies inside intron 5 of the *ADIPOR2 *locus and is not translated to amino acid. Nevertheless, such silent SNP could influence adiponectin receptor concentrations with unknown mechanisms. Our findings that genotypes of the rs767870 variant of the *ADIPOR2 *are associated with protein expression in circulating monocytes (the precursors of foam cells that populate atherosclerotic lesions), makes the hypothesis that *ADIPOR2 *could affect atherosclerosis through its protein expression attractive.

Monocytes play a pivotal role in inflammation and atherosclerosis. These are the sites in which adiponectin exerts its anti-inflammatory and anti-atherogenic effects, mainly by inhibiting the secretion of inflammatory cytokines from monocyte-derived macrophages, by preventing monocyte adhesion to endothelial cells and by suppressing macrophage-to-foam cell transformation [9,10]. Whilst initially our findings that associate increased atherosclerosis with higher *ADIPOR2 *expression in monocytes seem paradoxical, one could consider a compensatory up-regulation mechanism of *ADIPOR2 *expression to exist in individuals with advanced atherosclerosis in order to counteract their adverse metabolic and cardiovascular outcomes. Furthermore, upregulation of adiponectin receptors, which might reflect an increased need for adiponectin signalling has also been demonstrated in the adipose tissue of women with polycystic ovarian syndrome [19] and in the skeletal muscle of insulin resistant and type 2 diabetic subjects [16].

It is of particular interest that three different studies have identified the rs767870 variant of the *ADIPOR2*, as being associated either with triglyceride levels and fat accumulation or as a determinant of type 2 diabetes. More specifically, in a genetic association analysis in the French population, rs767870 was the only genetic marker associated with type 2 diabetes of the 12 *ADIPOR2 *SNPs studied [23]. The association remained significant in a meta-analysis study of three case-control studies. It was also the only one associated with liver fat content in the Finnish population [20]. Moreover, Richardson et al have demonstrated that homozygotes for the minor allele of rs767870 variant, were associated with lower triglyceride levels in insulin resistant Mexicans [28]. By contrast, this SNP was not associated with insulin resistance in another study in the German population [30].

However, our study findings should be interpreted bearing in mind some limitations. The number of our study subjects is generally regarded as small for genetic association studies. Nevertheless we found no major difference in the allelic frequencies of the rs767870 polymorphism in our Greek population, with the allelic frequencies in other European populations (HapMap-CEU). Nevertheless our findings must be confirmed on a larger sample and with other ethnicities.

## Conclusions

Our data provides evidence for the first time that the rs767870 variant of the *ADIPOR2 *could be a determinant of endothelial disturbance and early atherosclerosis in the Greek population, independent of insulin resistance, possibly through the elevated ADIPOR2 protein expression in circulating monocytes.

## Competing interests

The authors declare that they have no competing interests.

## Authors' contributions

IH: carried out the molecular genetics studies and drafted the manuscript, PCT: carried out the plasma analysis of adiponectin, designed the study, made the statistical analysis and revised the manuscript, II: participated in ultrasound studies and manuscript editing, AK and PM: contributed to data acquisition and manuscript editing, EM: carried out the immunofluorescence studies, EB: contributed to manuscript editing, JL and GD: conceived and designed the study, TE, DTK and SAR: revised and gave the final approval of the manuscript. All authors read and approved the final manuscript.
